# Heterologous Expression of *ATG8c* from Soybean Confers Tolerance to Nitrogen Deficiency and Increases Yield in Arabidopsis

**DOI:** 10.1371/journal.pone.0037217

**Published:** 2012-05-22

**Authors:** Tongmei Xia, Dong Xiao, Dong Liu, Wenting Chai, Qingqiu Gong, Ning Ning Wang

**Affiliations:** Department of Plant Biology and Ecology, College of Life Sciences, Nankai University, Tianjin, China; Kansas State University, United States of America

## Abstract

Nitrogen is an essential element for plant growth and yield. Improving Nitrogen Use Efficiency (NUE) of crops could potentially reduce the application of chemical fertilizer and alleviate environmental damage. To identify new NUE genes is therefore an important task in molecular breeding. Macroautophagy (autophagy) is an intracellular process in which damaged or obsolete cytoplasmic components are encapsulated in double membraned vesicles termed autophagosomes, then delivered to the vacuole for degradation and nutrient recycling. One of the core components of autophagosome formation, ATG8, has been shown to directly mediate autophagosome expansion, and the transcript of which is highly inducible upon starvation. Therefore, we postulated that certain homologs of *Saccharomyces cerevisiae* ATG8 (ScATG8) from crop species could have potential for NUE crop breeding. A soybean (*Glycine max*, cv. Zhonghuang-13) *ATG8*, *GmATG8c*, was selected from the 11 family members based on transcript analysis upon nitrogen deprivation. *GmATG8c* could partially complement the yeast *atg8* mutant. Constitutive expression of *GmATG8c* in soybean callus cells not only enhanced nitrogen starvation tolerance of the cells but accelerated the growth of the calli. Transgenic Arabidopsis over-expressing *GmATG8c* performed better under extended nitrogen and carbon starvation conditions. Meanwhile, under optimum growth conditions, the transgenic plants grew faster, bolted earlier, produced larger primary and axillary inflorescences, eventually produced more seeds than the wild-type. In average, the yield was improved by 12.9%. We conclude that *GmATG8c* may serve as an excellent candidate for breeding crops with enhanced NUE and better yield.

## Introduction

The vigor of plants is largely dependent on inorganic nitrogen (N), principally in the form of NO_3_^−^ and NH_4_^+^, and 85–90 million tons of nitrogenous fertilizers are applied worldwide annually [1], [2]. To maintain crop production, more and more commercial fertilizers are applied, generating enormous costs and resulting in severe environmental damage [3], [4], [5]. Therefore, breeding crops with high nitrogen use efficiency (NUE) and better yield has always been a major goal for breeders, and the identification of new potential NUE genes is important for scientists. Soybean is a major crop for protein and oil production globally. Though capable of biological N_2_ fixation when associated with *Rhizobia* bacteria, soybean acquires a large portion (from 40% to 75%) of nitrogen from soil, depending on the inorganic nitrogen content of soil [6]. It is estimated that, to produce 1,000 kg of soybean seeds, 70 to 90 kg of nitrogenous fertilizer needs to be applied [7]. Thus, it is critical to identify useful soybean genes for the development of new transgenic soybean cultivars with high NUE and better yield.

The nitrogen utilization of plants involves uptake, assimilation, translocation, and remobilization, in which nitrogen remobilization being the key step during seed maturation [8], [9], [10]. In Arabidopsis, for instance, nitrogen concentration (N%) in the dry remains was reported to be 4-fold lower at low nitrate supplies compared with high nitrate supplies, while nitrogen concentration in seeds barely changed [11]. In wheat (*Triticum aestivum*), remobilization contributes 69.8% to 88.8% to grain N content depending on cultivars [12]. The primary control for N filling in seeds is in the source organs [13], and N import into developing seeds is mainly derived from the amino acids produced by proteolysis of proteins synthesized before the onset of the reproductive phase [14]. RuBisCO, the most abundant soluble protein in green plants, as well as other photosynthesis-related proteins, is the major N source for remobilization. The main pathways for protein degradation include 26S proteasome/ubiquitin system, endoplasmic reticulum-associated degradation (ERAD), senescence-associated vesicles (SAV) and autophagy, leading to proteolytic degradation in the vacuole [9], [15]. Among them, autophagy has the largest capacity to degrade and recycle organelles and cytosolic macromolecules [16], [17], and could be an important source of N for seed production.

So far, most of our knowledge on autophagy has come from genetic studies in yeast (*Saccharomyces cerevisiae*), in which more than thirty AuTophaGy-related (ATG) genes have been identified [18], [19], [20]. Central to autophagy is the formation of the autophagosome, a double-membrane vesicle containing cargos ranging from damaged proteins to obsolete organelles to be degraded in the vacuole/lysosome. ATG8, a lipid-conjugated ubiquitin-like protein, whose transcript level is known to be greatly induced by starvation [21], serves as a scaffold for membrane expansion during autophagosome formation, and is the determinant of autophagosome size [22], [23]. ATG8 also participates in the cytoplasm-to-vacuole targeting (Cvt) pathway, a constitutive and specific form of autophagy, in which vacuolar hydrolases such as aminopeptidase 1 (APE1) and alpha-mannosidase (AMS1p) are selectively transported into the vacuole under normal growth condition [24], [25]. In both pathways, ATG8 binds to the cargo receptor ATG19, facilitating its localization to the pre-autophagosomal structure/phagophore assembly site (PAS), where the autophagic/Cvt transport vesicles are assembled [25], [26], [27].

In higher plants, homologous autophagic systems have been identified. *In vitro* reconstitution of the two conjugation systems (ATG5-ATG12 and ATG8-PE) has been successfully done, indicating that the mechanism of autophagy is conserved from yeasts to plants [28]. Analyses on the loss-of-function *atg* mutants revealed the involvement of autophagy in many physiological processes, including nutrient deficiency adaptation, disease resistance, innate immune response, and stress resistance [15], [29], [30], [31], [32], [33], [34], [35], [36]. An intriguing fact is that plants generally have many *ATG8*s; there are nine in Arabidopsis [29], [30], presumably five in maize [37], and five in rice [37]. The distinct expression patterns of individual *ATG8*s in Arabidopsis suggest that, apart from possible redundancy, at least some *ATG8*s may possess unique functions [33], [38]. Accordantly, Honig *et al* had found two plant specific AtATG8-interacting proteins (ATI1 and ATI2) that participate in the formation of a new type of compartment which was induced while exposure to carbon starvation [39]. They also showed that the constitutive expression of *AtATG8f* led to an increment of plant size, and that the transgenic plants became more tolerant to nitrogen and carbon starvation [40]. Given the specific function of *ATG8* and the expression characteristics of the plant *ATG8* gene families, we postulated that it is possible for us to identify at least one specific *ATG8* for the development of NUE crops. Furthermore, it has been shown that, under normal growth conditions, loss-of-function autophagy mutants such as *atg7*, *atg9*, and *atg10* produced less flowers, siliques, and seeds [29], [33], [38]. Whether constitutive expression of an *ATG* could improve seed yield is therefore worthy of exploring.

In order to screen for potential soybean NUE genes, our lab has previously set up a fast and effective soybean calli transformation system (patent pending), through which large amounts of uniformly transformed soybean cells could be obtained and analyzed on plates. Here, we firstly identified a soybean *ATG8* gene, namely *GmATG8c*, which is strongly induced by nitrogen starvation. We then confirmed its function as an ATG8 by yeast complementation. Constitutive expression of *GmATG8c* in soybean calli not only led to better tolerance towards nitrogen starvation, but accelerated calli growth under optimum growth conditions. Heterologous expression of *GmATG8c* in *Arabidopsis* led to better performance of the transgenic lines under both starvation conditions and normal growth condition. The transgenic lines had a better vegetative growth, entered the reproductive stage slightly earlier, and produced more flowers, siliques, and seeds. Our results implied that *GmATG8c* functions in soybean autophagy process, and that it may be an important candidate for breeding crops with enhanced NUE and better yield.

## Results

### Identification of *GmATG8c* as a Nitrogen Deficiency-inducible/responsive *ATG8* of Soybean

**Figure 1:**
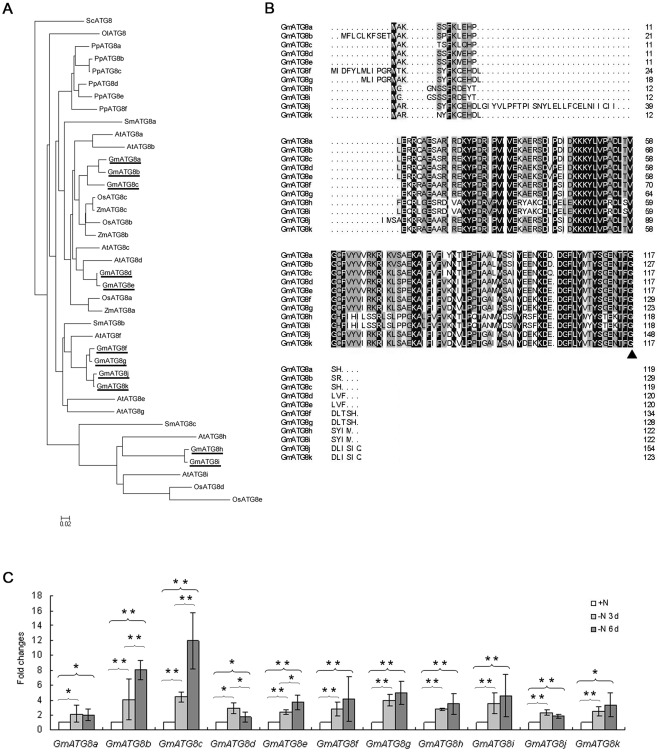
Soybean has eleven homologs of yeast ATG8. A, Phylogenetic tree of ATG8s in Glycine max (Gm), Arabidopsis thaliana (At), Saccharomyces cerevisiae (Sc), Selaginella moellendorffii (Sm), Physcomitrella patens (Pp), Ostreococcus lucimarinus (Ol), Oryza sativa (Os) and Zea mays (Zm). Deduced amino acid sequences were aligned by CLUSTALX and the phylogenetic tree was generated by the neighbour-joining method and displayed using MEGA5. GmATG8s were underlined. B, Deduced amino acid sequences of GmATG8c and alignment with ScATG8. Closed arrowhead indicates the C-terminal Glycine residue which is processed by Atg4 cysteine protease. C, Fold Changes of the transcript levels of soybean ATG8s in the primary leaves after nitrogen starvation. The average expression levels for the three biological replicates are shown. Significant differences between +N and –N for 3 days or 6 days, and between –N for 3 days and 6 days are labeled. All significant levels were calculated by t-test. *, significant (p<0.05); **, very significant (p<0.01).

Based on their sequence homology to the yeast (*Saccharomyces cerevisiae*) ATG8, eleven GmATG8s (see Table S1 for accession numbers) were identified from the soybean genome (Phytozome, http://www.phytozome.net/search.php) and named GmATG8a to GmATG8k, respectively (Fig. 1A). All GmATG8s possess a conserved Gly residue near their C-termini, which corresponds to the PE-acceptor site of yeast ATG8 (Fig. 1B). The GmATG8s share 42% to 94% sequence similarity with one another, and are all at least moderately similar to ScATG8, with 48% (GmATG8i) to 72% (GmATG8d) identical amino acid sequences (Table. S1). When a phylogenetic tree was built with ATG8 homologs from Arabidopsis, several major crop species including rice and maize, and lower green plants, the soybean ATG8s fall into three sub-groups (Fig. 1A). The first one is relatively close to ScATG8, and contains GmATG8a, b, c, d, and e. The second and third ones contain GmATG8f, g, j, and k; and GmATG8h and i, separately (Fig. 1A). The fern *Selaginella moellendorffii* possesses three ATG8s; each appeared in a sub-group (Fig. 1A).

**Table 1:** Bolting and flowering times of the wild-type and *35S:GmATG8c* transgenic Arabidopsis.

	WT	L-2	L-3	L-4
bolting time (d)	26.9±0.48	25.48±0.34*	25.56±0.29*	24.84±0.28**
flowering time (d)	29.55±0.46	28.32±0.42*	28.33±0.29*	27.65±0.32**

* p<0.05 (t-test); significant difference from the wild-type (WT).

** p<0.01 (t-test); very significant difference from the wild-type (WT).

The two traits were scored as days after sowing (DAS).

Similar to their homolog in yeast, transcript levels of some specific Arabidopsis *ATG8*s have been shown to be induced by nutrient deprivation [30], [41], [42]. We examined whether the transcript levels of *GmATG8*s could be induced by nitrogen starvation by real-time RT-PCR (Fig. 1C). When the soybean seedlings (8-day-old) grown hydroponically were deprived of nitrogen for 3 days, the transcript levels of all *GmATG8*s in the primary leaves were all slightly induced (p<0.05) (Fig. 1C). When the time of N-starvation was prolonged to 6 days, expression of some *GmATG8*s was further induced, with *GmATG8c* peaked at 12 fold (Fig. 1C). The results indicated that the *GmATG8c* was a potential soybean functional *ATG8* gene in response to nitrogen starvation. GmATG8c encodes a soluble protein of 119 amino acids, with a predicted pI of 9.16 (http://web.expasy.org/compute_pi/) [43]. A 3D model for GmATG8c was constructed with Phyre 2 (www.sbg.bio.ic.ac.uk/phyre2) [44] to reveal its striking resemblance to the yeast, protist and mamalian ATG8 homologs; the protein is composed of an N-terminal domain containing two α-helices and a C-terminal ubiquitin-like domain (Fig. S1).

### Partial Complementation of *Saccharomyces cerevisiae atg8* Mutant by *GmATG8c*

**Figure 2:**
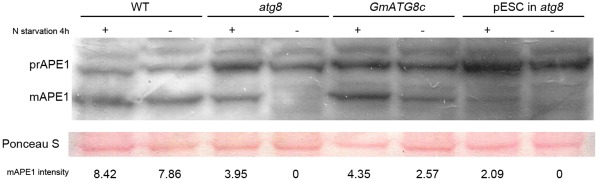
Functional complementation of a yeast *atg8* mutant by GmATG8c. Cultures grown to mid-log phase in YPD were harvested or shifted to YPD without nitrogen for 4 hours and then harvested for protein extraction (3OD_600_ cells each). Proteins were then resolved by SDS-PAGE followed by immunoblotting with anti-APE1 antibody. Intensities of band signals of mAPE1 were quantified using the software Quantity One (Bio-Rad). Five independent replicates were done to give the typical results shown here. WT: TN124; *atg8*: TN124 *atg8Δ:KAN URA3 TRP1*; *GmATG8c*: *atg8* carrying *pADH1-GmATG8c;* pESC in *atg8*: *atg8* carrying *pADH1* only (on pESC).

In yeast, as a cargo of Cvt/autophagy pathway, the precursor amino-peptidase 1 (prAPE1) is incorporated into Cvt vesicles/autophagic bodies and delivered to the vacuole for processing into mature APE1 (mAPE1), making it a marker for Cvt and autophagy pathways [45], [46]. To verify the function of GmATG8c, we carried out a functional complementation experiment, in which the protein levels of both prAPE1 and mAPE1 were monitored. Upon nitrogen starvation, mAPE1 accumulated in wild-type yeast cells which suggested pAPE1 protein has been delivered to the vacuole, and that processing of pAPE1 into mAPE1 occurred (Fig. 2). *atg8* cells transformed with *pADH:GmATG8c* also accumulated mAPE1, albeit to a lesser extent than the wild-type (Fig. 2). In contrast, in *atg8* cells or *atg8* cells transformed with the vector containing the *ADH* promoter only, no mAPE1 was detected under nitrogen rich condition, and mAPE1 were barely detectable under nitrogen starvation condition (Fig. 2). The result hence confirmed the role of GmATG8c as an ATG8.

### Constitutive Expression of *GmATG8c* Enhanced the Tolerance of Soybean Calli to Nitrogen Limitation

**Figure 3:**
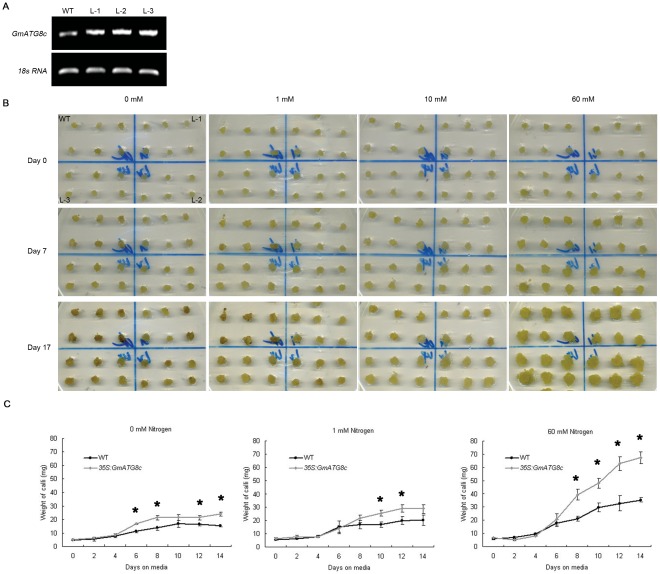
Constitutive expression of *GmATG8c* enhanced the tolerance of soybean calli to nitrogen limitation conditions. A, The constitutive expression of *GmATG8c* in transgenic calli lines was confirmed by semi-quantitative RT-PCR. WT: wild type calli. L-1 to L-3: three *35S:GmATG8c* transgenic calli lines. B, Morphology of *GmATG8c* over-expressing calli and the wild-type cultivated on MS medium with indicated nitrogen concentration over indicated period of time. The calli were placed on the medium all in the same order as marked in the upper left panel (0 mM, Day 0). C, Growth curves of *GmATG8c* over-expressing calli and those of the wild-type on medium with full (60 mM), low (1 mM), and no (0 mM) nitrogen over 14 days. Results are the mean ± SE for three biological replicates.*, p<0.05 (t-test); significant difference from the wild-type (WT).

We then evaluated the NUE potential of *GmATG8c* at the cellular level. *GmATG8c* was constitutively expressed under the control of the *CaMV 35S* promoter in soybean calli. The over-expression of *GmATG8c* in three homozygous callus lines was confirmed by semi-quantitative RT-PCR (Fig. 3A). Transgenic and wild-type calli of similar size were transferred to MS medium with 60 mM N (sufficient nitrogen, as control), 10 mM N and 1 mM N (low nitrogen) or without nitrogen. The status of the calli was evaluated by their weight and browning rate [47]. On day 7, the wild-type calli grown on medium with 1 mM N or no N began to turn brown, while the transgenic calli remained transparent (Fig. 3B). On day 17, the wild-type calli grown on 1 mM N had turned brown more severely, while the transgenic calli remained healthy (Fig. 3B). Furthermore, nitrogen deficiency resulted in growth retardation in the wild-type earlier than in the transgenic calli (Fig. 3B). A typical transgenic line, together with the wild-type, was weighed daily to track the growth of the calli. Under all conditions, the *GmATG8c* over-expressing calli grew much faster and became significantly heavier than the wild-type (Fig. 3C). The results further confirmed that over-expression of *GmATG8c* confers nitrogen deficiency tolerance at the cellular level.

### *GmATG8c* Enhanced Tolerance to Nitrogen Starvation in Transgenic Arabidopsis

**Figure 4:**
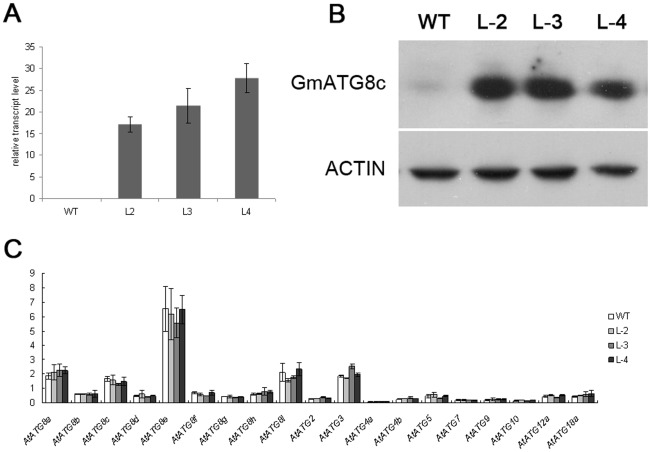
Heterologous expression of *GmATG8c* in Arabidopsis. A, Quantitative RT-PCR analysis of *GmATG8c* transcript in 5-day-old seedlings of the wild-type (WT) and *35S:GmATG8c* lines (L-2 to L-4). B, Immunoblot analysis of the accumulation of GmATG8c in 5-day-old seedlings of the wild-type (WT) and *35S:GmATG8c* lines (L-2 to L-4) with a polyclonal anti-GmATG8c antibody. Equal protein loads were confirmed by immunoblot analysis with an anti-Actin antibody. C, Real-time RT-PCR analysis of the transcript levels of *AtATGs* in 5-day-old seedlings of the wild-type and *35S:GmATG8c* lines. The average expression levels for the three biological replicates are shown (p>0.05). Heterologous expression of *GmATG8c* appeared to have no effect on the transcript levels of the endogenous *AtATG8*s.

We then evaluated the NUE potential of *GmATG8c* in the model plant Arabidopsis. *35S:GmATG8c* was introduced into Arabidopsis by floral dipping. The heterologous expression of *GmATG8c* in three T_3_ homozygous transgenic lines was confirmed through both quantitative RT-PCR (Fig. 4A) and western blotting (Fig. 4B, S2). Real-time RT-PCR showed that the transcript levels of the endogenous *AtATG*s were unchanged in the transgenic lines (Fig. 4C).

**Figure 5:**
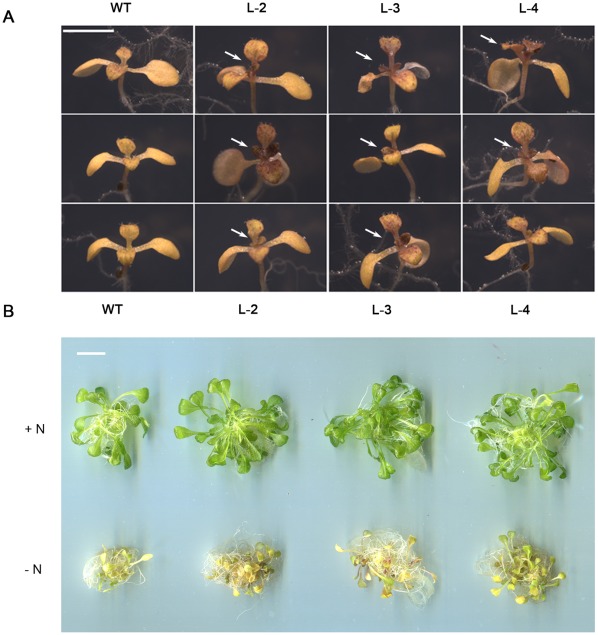
*35S:GmATG8c* seedlings were more tolerant to nitrogen starvation than the wild-type. A, Five-day-old seedlings of the wild-type (WT) and *35S:GmATG8c* lines were transferred to 1/2MS medium without nitrogen and starved for 21 days under a long-day photoperiod, then photographed. The white arrowheads indicate the newly emerged true leaves. Scale bar  = 50 µm. B, Seedlings (n = 10) were grown for 1 week on 1/2MS+N liquid medium and then transferred to N-rich (+N) or N-deficient (–N) media for 4 days. Scale bar  = 1 cm. Results from one out of five biological replicates were shown.

Nitrogen starvation tolerance was evaluated in both seedlings and adult plants. Five-day-old seedlings of the wild-type and transgenic lines, similar in sizes, were transferred to 1/2 MS medium without nitrogen. Within three weeks, the difference in sizes became clearly visible. Besides, all transgenic lines produced in average two more true leaves than the wild-type (p<0.05) (Fig. 5A). When grown under continuous light in liquid medium free of nitrogen, the wild-type plants ceased growing, whereas the transgenic plants continued to grow, only at a reduced rate (Fig. 5B). The phenotypes suggested that the transgenic seedlings did endure better under the nitrogen-deficient condition.

**Figure 6:**
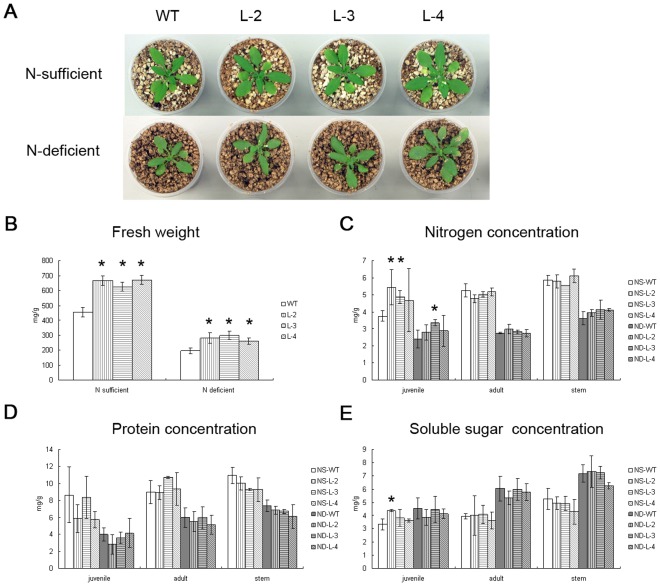
*GmATG8c* confers nitrogen starvation tolerance to adult plants. Wild-type and *35S:GmATG8c* plants were grown hydroponically in a mixture of pearlite/vermiculite irrigated every other day with half Hoagland’s until bolting (23 d after sowing), and then maintained in either the same nitrogen-rich solution (N sufficient) or transferred to nitrogen-free solution (N deficient) for another 5 days. A, Top view of the wild-type and *35S:GmATG8c* lines without (upper panel) and with (lower panel) 5 days of nitrogen starvation. B, The fresh weights of the wild-type and transgenic lines. C, Nitrogen contents in juvenile/adult rosette leaves and stems of the wild-type and *35S:GmATG8c* lines. D, Protein contents in the materials. E, Soluble sugar contents in the materials. Representative data from one out of three biological replicates are shown. NS: N sufficient, ND: N deficient. Data shown are the means ±SD of one representative biological replicate (n = 21) out of three.^*^, p<0.05 (t-test); significant difference from the wild-type (WT).

To evaluate the tolerance of nitrogen starvation in the adult plants, we set up a hydroponic system similar to a previous study [48]. Nine-day-old seedlings grown on 1/2 MS medium were transferred to vermiculite and perlite (1∶1, v/v) supplied with 1/2 Hoagland’s solution and let grown to bolting (23 days after sowing, DAS), then transferred to either 1/2 Hoagland’s solution or nitrogen-free solution and let grown for another 5 days. On 28 DAS, all three transgenic lines had slightly, yet significantly larger rosettes than the wild-type under both nitrogen-sufficient (p  = 0.022, 0.029, 0.012) and nitrogen-deficient condition (p = 0.010, 0.003, 0.001) (Fig. 6A and Table. S3). Meanwhile, all plants were harvested and weighed. Under both the conditions with or without nitrogen starvation, all three *GmATG8c* over-expressing lines weighed significantly more than the wild-type (Fig. 6B). Contents of nitrogen, protein, and soluble sugar were measured in control and starved plants. As expected, nitrogen contents and protein levels in all materials decreased in the nitrogen-deprived wild-type and transgenic lines (Fig. 6C, D). The transgenic lines accumulated more nitrogen in their rosette leaves and stems (Fig. S3). Specifically, in the juvenile rosette leaves (leaves 3 to 6), the nitrogen concentration (mg/g of fresh weight) in the transgenic lines was higher than that of the wild-type (Fig. 6C). The adult rosette leaves (leaves 7 to 10) and the stems of the transgenic plants had similar nitrogen contents with the wild-type (Fig. 6C).

Under the nitrogen-sufficient condition, the protein concentrations in the juvenile rosette leaves of the transgenic plants seemed lower than those of the wild-type, but showed no significant difference by student’ s t-test (p>0.05) (Fig. 6D). In both juvenile and adult rosette leaves, the protein concentrations were similar between the wild-type and transgenic plants under the nitrogen-deficient condition (Fig. 6D).

As expected, more soluble sugar accumulated in the nitrogen-deprived wild-type and transgenic plants (Fig. 6E). The soluble sugar concentrations in the transgenic plants generally exhibited no difference from those of the wild-type in all the materials under the same condition (Fig. 6E).

### *GmATG8c* Over-expressing Arabidopsis Adapted Better to Carbon (C-) Starvation than the Wild-type

**Figure 7:**
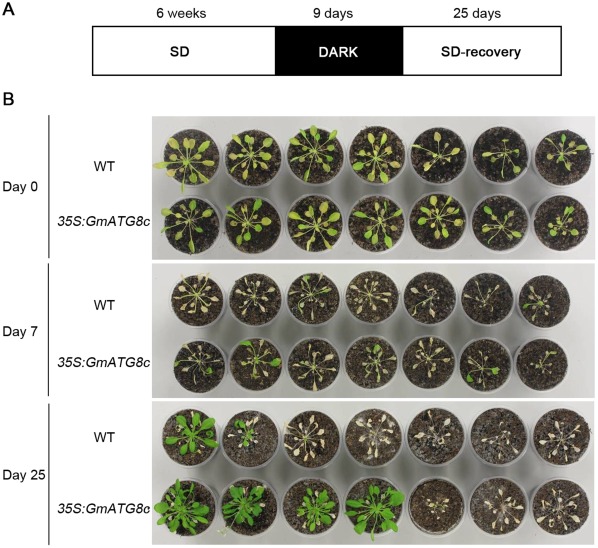
Enhanced tolerance of *35S:GmATG8c* to carbon-limiting conditions induced by extended darkness. A, Diagram of treatment. Six-week-old plants were grown under a short-day (SD) photoperiod, transferred to darkness for 9 days, and then transferred back to the short-day photoperiod for recovery. B, The upper panel shows plants immediately after dark treatment; the middle panel shows plants after 7-day recovery; and the bottom panel shows plants after 25-day recovery. Data from one representative line (L-4) out of four lines analyzed are shown.

Previous studies have shown that the Arabidopsis *atg* mutants were generally more sensitive to carbon limitation conditions [32], [33], [38]. To see if *GmATG8c* over-expressing Arabidopsis could better survive carbon limitation, seedlings of a typical transgenic line (L-4) and the wild-type were grown on 1/2 MS to stage 1.02 [49], transferred to soil and let grown for 33 more days under short-day condition [50], [51], kept in dark for 9 days, then allowed to recover under the same short-day condition (Fig. 7A). A larger portion of *35S:GmATG8c* transgenic plants survived, and resumed growth earlier than the wild-type (Fig. 7B). Therefore, *GmATG8c* indeed conferred carbon starvation tolerance to Arabidopsis.

### Constitutive Expression of *GmATG8c* Promotes Growth and Increases Yield in Arabidopsis

**Figure 8:**
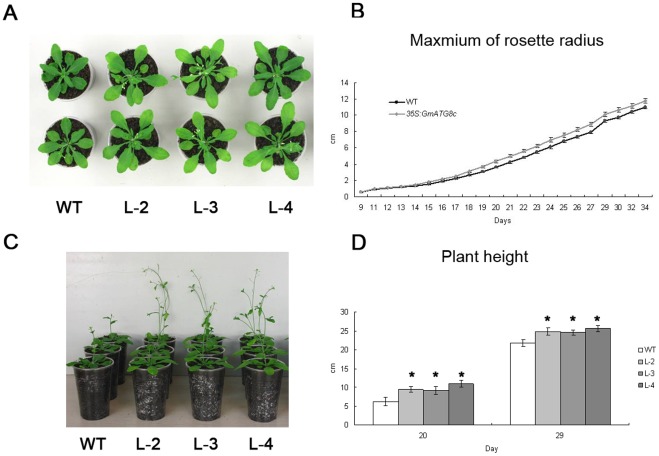
Heterologous expression of *GmATG8c* promotes growth in Arabidopsis. Nine-day-old seedlings were transferred to soil and cultivated under a long-day photoperiod. The radius of rosette and the plant height were recorded daily. A, Top view of 4-week-old representative wild-type (WT) and *35S*:*GmATG8c* lines (L-2, L-3, L-4) grown under a long-day photoperiod. B, Growth curves of the rosette of the wild-type and one representative transgenic line (L-4). C, Side view of 5-week-old representative wild-type (WT) and *35S*:*GmATG8c* lines (L-2, L-3, L-4) grown under a long-day photoperiod. D, The plant height of the wild-type and transgenic lines. Data shown are the means ±SE of one representative biological replicate (n = 24) out of three. X-axis represents the days after sowing (DAS).

We have noticed from the nitrogen starvation experiments that the transgenic plants grew faster than the wild-type (Fig. 6A). Therefore, the growth parameters of the transgenic plants and the wild-type under normal growth condition under long-day photoperiods were recorded daily over a period of 8 weeks (Fig. 8). The rosettes of *GmATG8c* over-expressing plants reached larger sizes upon bolting (Fig. 8A) and grew faster than the wild-type (Fig. 8B). The plant heights of transgenic plants were higher than the wild-type plants at the time points we observed (Fig. 8C, D). All the transgenic lines bolted and flowered slightly earlier than the wild-type (Table 1). These data indicated that the over-expression of *GmATG8c* could promote vegetative growth of Arabidopsis, and facilitate the transition into reproductive growth.

**Figure 9:**
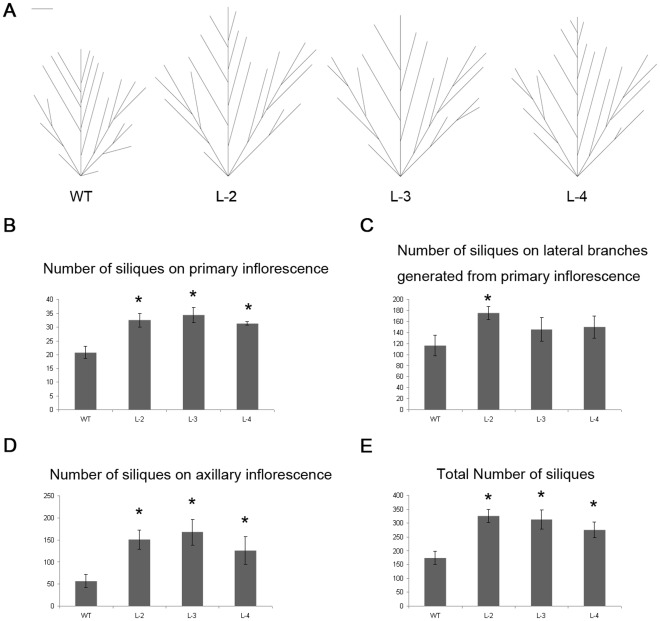
Phenotypic characterization and yield-related characters of the *35S:GmATG8c* transgenic Arabidopsis. A, Schematic diagrams of branching patterns of wild-type plants (WT) and *GmATG8c* over-expressing lines (L-2 to L-4). Bar  = 5 cm. B, The number of siliques on the primary inflorescence of the wild-type and *35S:GmATG8c* transgenic lines. C, The number of siliques on lateral branches generated from the primary inflorescence. D, The number of siliques on axillary inflorescence of the wild-type and *35S:GmATG8c* transgenic lines. E, Total number of siliques of the wild-type and *35S:GmATG8c* transgenic lines. Data shown are the means ±SE of one representative biological replicate (n = 10) out of two. ^*^, p<0.05 (t-test); significant difference from the wild-type (WT).

We then analyzed the reproductive phase of the plants in more details (Fig. 9). Branching patterns were dissected in seven-week-old wild-type and transgenic lines, and it became clear that although the plant architecture has not changed, the transgenic lines did have larger primary and axillary inflorescences that looked more robust (Fig. 9A, S4). The primary inflorescence (without the lateral branches) of the transgenic lines produced significantly more fertile siliques than the wild-type (Fig. 9B), which is consistent with our observation that the transgenic lines entered the reproductive phase slightly earlier (Table 1). The numbers of siliques of transgenic plants on the primary inflorescence and the axillary inflorescences were larger than the wild-type (Fig. 9C, D). Overall, all three transgenic lines produced significantly more siliques than the wild-type (Fig. 9E).

To further evaluate the potential of *GmATG8c* in yield increment, four transgenic lines (L1-4) were grown to stage 9.70 [49] for seed harvest. All seeds from individual plants were harvested and weighed. All four transgenic lines evaluated had higher yields than the wild-type (Table 2). One of the lines was subjected to further analysis on the reason for the increment of yield. The thousand grain weight was not different from that of the wild-type (Table 3). The total number of seeds per silique, however, differed slightly (by two seeds) but significantly from that of the wild-type (Table 3). The total number of siliques was 12.7% more than the wild-type, which may have a major contribution to the increment of yield in *GmATG8c* over-expressing plants (Table 3).

**Table 2:** The yields of *35S:GmATG8c* transgenic lines and those of the wild-type.

	WT	L-1	L-2	L-3	L-4
Yield (mg)	296.93±9.59	343.15±19.16	313.51±5.78	321.36±14.84	362.78±14.43*
Percentage increased	–	15.6%	5.6%	8.2%	22.2%

All seeds produced by individual plants were harvested and weighed.

* p<0.05 (t-test); significant difference from the wild-type (WT).

**Table 3:** Yield related characteristics of the wild-type and *35S:GmATG8c* transgenic Arabidopsis.

	WT	*35S:GmATG8c*	Increment of percentage
Total number of siliques	387±33	436±17*	12.7%
Total number of seeds per silique	58.36±0.87	60.36±0.81*	3.42%
Yield per plant (mg)	272.98±16.37	309.87±17.84*	13.51%
Thousand grain weight (mg)	15.11±0.57	15.68±0.51	–

* p<0.05 (t-test); significant difference from the wild-type (WT).

To further explore the yield increment potential of *GmATG8c*, we also transformed tomato (cv. Micro-Tom), a model plant for fruit bearing, with *35S:GmATG8c*. Similar to our observations in transgenic Arabidopsis, *35S:GmATG8c* transgenic tomato also grew faster and entered reproductive phase earlier than the wild-type, and produced more fruits (Fig. S5, Table S4).

Based on our observations, we concluded that *GmATG8c* not only has NUE potential but also has potential for yield increment, and may be an excellent target gene for breeding better crops.

## Discussion

Though autophagy has been established as a ubiquitous intracellular degradation pathway of eukaryotic cells, much of the detailed process, and even components of autophagy, remains elusive in plants, especially in crop species. We identified eleven soybean *ATG8* genes through sequence analysis. It is well known that a number of ATG8 isoforms exist in plants and mammals, and the existence of an *ATG8* gene family may reflect the existence of specific functions of individual *ATG8*s, as have been suggested by other studies [29], [30], [38], [52]. When making a phylogenetic tree for ATG8 homologs in crops and lower plants, we noticed two interesting facts. Firstly, all six ATG8s of the moss *Physcomitrella patens* were clustered together to form a branch relatively close to the AtATG8a and GmATG8c (Fig. 1A). Secondly, all other ATG8s formed two more sub-groups represented by AtATG8f and the longer ATG8s, separately (Fig. 1A). The branching pattern of the phylogenetic tree suggests that a major functional specialization event of plant ATG8s may have happened when tracheophytes firstly emerged.

Based on the hypothesis that, similar to the yeast *ATG8*, the transcript level of a functional plant *ATG8* may be highly induced by nitrogen deprivation, we searched for such an *ATG8* homolog from soybean. Out of the eleven *ATG8*s, *GmATG8c* showed the distinctively high level of induction (more than ten folds) under nitrogen deprivation (Fig. 1C). We then confirmed the biochemical function of GmATG8c as an ortholog of ScATG8 by yeast complementation in a similar fashion to a previous study, in which AtATG6 was confirmed as a functional ortholog of ScATG6 [53] (Fig. 2).

We further verified the physiological function and NUE potential of GmATG8c at both cellular and whole-plant level. It has been shown in maize that, during nitrogen and carbon limitations, *ATG* transcripts and ATG8-PE accumulates [37]. Such observation indicates that autophagy may play a key role in nutrient remobilization. Our hypothesis was that an extra dose of a nitrogen limitation-inducible ATG8 may confer nitrogen-limitation tolerance by promoting autophagy, and thus nitrogen remobilization, more efficiently when necessary.

Indeed, the transgenic lines excelled in many ways. Not only did they survive extended period of carbon or nitrogen starvation, but promptly recovered and resumed growth when moved back to normal growth conditions. It is noteworthy that the transgenic lines generally produced more leaves when starved (Fig. 5A, 6A) or moved back to optimum conditions (Fig. 7B), suggesting that the ectopic expression of *GmATG8c* contributes to the fitness and survival of the shoot apical meristem. Two previous studies had suggested the involvement of autophagy in the activity and maintenance of root apical meristem: over-expression of *AtATG8f* resulted in elevated sensitivity of root growth towards exogenous cytokinin in Arabidopsis [40], and an *atg5* mutant of Arabidopsis failed to keep its root apical meristem alive upon phosphorus limitation [54]. Therefore, the positive function of autophagy in the maintenance of meristem activity could be essential for the adaptation of plants towards nutrient limitation.

Interestingly, under optimum growth conditions, no change in nitrogen concentrations was observed in the adult rosette leaves or stems of the transgenic lines from those of the wild-type (Fig. 6C), whereas the growth of the plants appeared accelerated [Fig. 6A, 6B, 8A, 8B, Table S3]. Such observations were consistent with our hypothesis that nitrogen remobilization was more efficient in *35S:GmATG8c*. Possibly a result of better vegetative growth, the transgenic lines also had a longer period of reproductive growth, and produced more siliques and seeds. The yield of transgenic lines were improved (Table 2) not for the thousand grain weight, but for the total seed number, in which the increment of siliques contributed more compared with the increment of number of seeds per silique (Table 3). It is known that grain yield is based both on the nitrogen uptake from soil and on the nitrogen remobilization during seed maturation [12]. The organic nitrogen supply from source leaves during grain filling is of particular importance [55], [56], since it contributes to plant N economy and limits exogenous N demand after flowering [9]. Since autophagic recycling has been suggested to be required for N and C mobilization [9], [57], the yield increment in the transgenic lines thus could be due to an enhanced autophagic recycling ability which led to more efficient nutrient remobilization from the rosette into the reproductive organs.

In order to improve the NUE and yield of crops, many genes have been analyzed mainly through transgenic (over-expression) studies. Traditional approach has focused on the essential genes in nitrogen uptake and assimilation, including *NRT*s (*nitrate transporters*), *NR* (*nitrate reductase*), *NiR* (*nitrite reductase*), *GS* (*glutamine synthesis*), and *GOGAT* (*glutamate synthesis*) [58]. Despite the great efforts, the corresponding transgenic plants have shown little improvement in NUE and yield [58], except in one case: the over-expression of *NADH-GOGAT* (*NADH-dependent glutamate synthesis* ) of rice enhanced grain filling and increased grain weight [59]. Therefore, new pathways should be explored for transgenic purposes. Since autophagy is one of the main pathways for protein degradation, and very likely contributes to the nitrogen pool for seed production, autophagy-related genes may have NUE potential and could probably improve the yield. Previous reports and our study have seen the reduced fitness and fertility of several *atg* mutants, including *atg5* (data not shown), *atg7*, *atg9, atg10* [29], [33], [38], and our study showed that the over-expression of a specific ATG8 could make soybean cells and Arabidopsis plants grow better under both nitrogen-rich and nitrogen-poor conditions. Clearly, autophagy is a pathway with NUE potentials. It is noteworthy that our strategy accord with one of the strategies proposed in [60], which is to modify C- and N- allocation and to elevate the strength of the sink [60].

GFP-ATG8 has been widely used as a marker of autophagy [30], [32], [61], [62]. Nevertheless, the current work, together with a previous study [40], demonstrates that the over-expression of plant *ATG8s* could have impact on the response to nutrient starvation and growth at both the cellular and the whole plant level. Therefore, we suggest that caution should be taken when using GFP-ATG8 driven by strong promoters as a marker for autophagy in plants, especially when studying plant growth and development.

In summary, our study has linked autophagy to plant nitrogen homeostasis/distribution as well as yield formation, and has identified a new node with great potential to be targeted in molecular/transgenic breeding to enhance NUE and improve yield in crop species. Further study will be carried out to elucidate the detailed molecular mechanisms through which *GmATG8c* promotes NUE and increases yield.

## Materials and Methods

### Sequence Analysis of ATG8 Proteins

Deduced ATG8 amino acid sequences of *Glycine max* (Gm), *Physcomitrella patens* (Pp) and *Selaginella moellendorffii* (Sm) were identified by searching the corresponding genomic sequence (http://www.phytozome.net/) using *Saccharomyces cerevisiae* ATG8 as the query. The ATG8 amino acid sequence of *Ostreococcus lucimarinus* (Ol) was identified by searching the *Ostreococcus lucimarinus* Assembled RefSeq Genomes (http://blast.ncbi.nlm.nih.gov/) using ScATG8 as the query. The ATG8s serial numbers of *Oryza sativa* (Os) and *Zea mays* (Zm) were used as described [37]. OsATG8s and ZmATG8s were identified as follows, with the corresponding loci in parentheses; OsATG8a (NP_001059767), OsATG8b (NP_001053929), OsATG8c (NP_001061171), OsATG8d (NP_001065220), OsATG8e (NP_001065220), ZmATG8a (NP_001137492), ZmATG8b (NP_001137493), and ZmATG8c (NP_001137496). The serial numbers of GmATG8s and SmATG8s were made according to their relationship with Arabidopsis ATG8s. The Phytozome ID and GenBank accession numbers of the corresponding GmATG8s and SmATG8s can be found in Table S1.

Amino acid sequences were aligned using CLUSTALX2 (http://www.clustal.org/) [63]. The phylogenetic tree was generated in CLUSTALX2 by the neighbour-joining method and a thousand replicates and displayed using MEGA5 (http://www.megasoftware.net/) [64].

The 3D model for GmATG8c was constructed with Phyre 2 (www.sbg.bio.ic.ac.uk/phyre2 ) [44] and compared with the model of *Homo sapiens* GABARAP (HsGABARAP), *Saccharomyces cerevisiae* ATG8 (ScATG8) and *Trypanosoma brucei* ATG8 (TbATG8).

### Expression Analysis of *GmATG8* Genes Responsive to N Limitation

Real-time RT-PCR was used to compare the expression of *GmATG8*s induced by nitrogen starvation. Four-day-old soybean seedlings were transferred to half Hoagland solution, and then transferred to N-deficient half Hoagland with the cotyledons cut off, when the primary leaves unfolded. After 3 d and 6 d N-limitation treatment, the primary leaves were harvested for gene expression analysis. The half Hoagland hydroponic Solution (pH 5.7), contained 3 mM KNO_3_, 0.5 mM sodium biphosphate buffer (pH 5.7), 2.0 mM Ca(NO_3_)_2_, 1.0 mM MgSO_4_, 0.25 mM (NH_4_)_2_SO_4_, 50 µM Fe-EDTA, 0.7 mM H_3_BO_4_, 170 µM MnCl_2_, 2.0 µM Na_2_MoO_4_, 100 µM NaCl, 5.0 µM CuSO_4_, 10 µM ZnSO_4_, and 0.1 µM CoCl_2_. The nitrogen-free solution was prepared by replacing KNO_3_ and Ca(NO_3_)_2_ with KCl and CaCl_2_, respectively. Three biological replicates, represented by three batches of plants grown at different time, were analyzed as follows.

RNA extraction and cDNA synthesis were done as described [65]. Total RNA was extracted, and residual genomic DNA was digested with RNase-free DNase I. The absence of genomic DNA was confirmed with a control PCR. First strand cDNA was synthesized from 2.0 µg of total RNA using AMV reverse transcriptase (Promega, Madison, WI, USA). cDNA samples diluted for 10 folds were used as templates.

Real-time RT-PCR analysis was performed using SYBR Green Perfect mix (TaKaRa, Dalian, China) on an iQ5 (Bio-Rad, California, USA). All reactions were performed under the following conditions: 95°C for 2 min; 40 cycles of 95°C for 10 s and 60°C for 30 s. All reactions were done at least in triplicates. *Actin11* was used as an internal control. All primers used are listed in Table S2. Results from one out of three biological replicates were shown.

### Functional Complementary Test in Yeast *atg8* Mutant

The plasmid *pESC-URA* was constructed by ligating the corresponding *GmATG8c* ORF fragments and *ScADH1* promoter fragments into *Spe* I and *Bgl* II?sites of *pESC-URA* and *EcoR* I?and *Spe* I sites of *pESC-URA* respectively. In addition, the plasmid *pESC-URA* was constructed by ligating the corresponding *ScADH1* promoter fragments into *EcoR* I?and *Spe* I sites of *pESC-URA* alone for control. The primers used are listed in Table S2.

The plasmids *pESC-URA* with *ADH1:GmATG8c* or *ADH1* promoter alone were introduced in the *atg8* mutant (TN124 *atg8Δ:KAN URA3 TRP1)* respectively. Three OD_600_ cell extracts from mid-log phase growing cells or 4 hours nitrogen-starved cells were resolved by SDS-PAGE followed by immunoblotting with anti-APE1 antibody, and equal transfer to the membrane was confirmed by Ponceau S staining (Sigma, USA). Preparation of cell extracts and immunoblot analyses were performed as described [66]. Intensities of band signals of mAPE1 were quantified using the software Quantity One (Bio-Rad). Five independent replicates were done to give the typical results shown here. The wild-type yeast (strain TN124) [67], *atg8* yeast mutant (TN124 *atg8Δ:KAN URA3 TRP1*) and anti-APE1 antibody were gifts from Dr. Zhiping Xie (Nankai University, China).

### Construction of Binary Over-expression Vector of *GmATG8c* Gene and Plant Transformation

The complete coding region of *GmATG8c* was amplified by PCR using a pair of primers F (*Nco* I site was introduced) and R (*BstE* II site was introduced) with full-length cDNA of soybean leaves as the template. The specific PCR fragment was then digested and ligated into binary vector *pCAMBIA1301* between *Nco* I and *BstE* II sites, resulting in the *35S:GmATG8c* fusion gene. The authenticity of the fusion construct was confirmed by DNA sequencing. Agrobacterium-mediated floral dipping method [68] was used for Arabidopsis transformation. The primers used are listed in Table S2.

T_1_ transformants of Arabidopsis were screened on half-strength Murashige and Skoog (1/2 MS) medium containing 30 µg/ml hygromycin. Within 2 weeks, seedlings with green true leaves were identified as transformants and transferred to soil, then verified by PCR and RT-PCR. Seeds collected from individual T_1_ tranformants were scored for segregation on hygromycin to determine the number of T-DNA insertion. Five homozygous T_2_ lines were obtained, and the ectopic expression of *GmATG8c* were verified by real-time PCR (data not shown). For this study, three homozygous lines were analyzed in details. The transcript levels of the endogenous autophagy-related genes were analyzed through real-time RT-PCR. Primers used are listed in Table S2 or as described [30], [69].

The soybean hypocotyl segments were inoculated with *Agrobacterium*, co-cultivated in darkness for 3 days, and then transferred to callus induction medium (Liu, et al, unpublished) for calli induction. The callus tissues that were induced at both the acropetal and basipetal ends were excised from the hypocotyl segment explants and transferred to callus screening medium (Liu, et al, unpublished) for further selection. Over-expression of *GmATG8c* was confirmed by RT-PCR. Three independent transgenic lines were analyzed.

The method for generation of *35S:GmATG8c* transgenic tomato was described in Methods S1.

### Plant Protein Isolation and Immunoblot Analysis

Total proteins were extracted in extraction buffer as described [65], and the homogenates were centrifuged at 12,000 rpm for 15 min at 4°C. Protein concentration was determined with the Bradford method [70]. The full length GmATG8c was used to raise a polyclonal antibody (Abmart, Shanghai China). 20 µg protein extracts were resolved by Tricine-SDS-PAGE followed by immunoblotting with anti-GmATG8c antibody and anti-Actin (Santa Cruz Biotechnology, USA). Five independent replicates were done to give the typical results shown here.

### Plant Growth Conditions and Phenotypic Analysis

Arabidopsis seeds (Columbia-0) were surface-sterilized in 1% (v/v) sodium hypochlorite for 2 min, washed with sterilized water for 10 times, stratified in water at 4°C for 2 days in the dark, and grown on half-strength Murashige and Skoog (1/2 MS) medium (0.8% agar (w:v), pH 5.7, 1% sucrose). The plates were incubated in a plant growth chamber (22°C/19°C, 16 h light/8 h dark, with a photosynthetic photon ﬂux density (PPFD) of 80 µmol m^−2^ s^−1^). Plants were transferred to soil after 9 days to analyze the basal phenotype in the sufficient nutrient condition, and the maximum rosette radius and the plant height were recorded every day. Seeds were harvested individually at the growth stage 9.7 [49] and weighed. Three biological repeats were done to give the typical results shown here.

To analyze and illustrate the plant architecture, the numbers of branches of 7-week-old wild-type and *35S:GmATG8c* lines were counted, and the lengths of each branch were measured. To properly describe the branching patterns, individual branches that have been produced by over 70% of plants were accepted as present. Then the average lengths of individual branches were calculated for illustration using Adobe Illustrator (CS5).

To study the responses to low nitrogen treatment, wild-type and *GmATG8c* constitutive-expressing calli were grown on MS medium with 0 mM, 1 mM, 5 mM and 60 mM nitrogen for up to a month under a long-day photoperiod (16 h/8 h) at 25°C. Three biological repeats of three transgenic lines with different culture runs were done.

To study the response of the wild-type and *35S:GmATG8c* transgenic Arabidopsis towards nitrogen starvation, young seedlings and adult plants were tested separately. Five-day-old seedlings grown in the long-day photoperiod (16 h/8 h)were transferred to N-deficient agar medium with 1% Suc: Murashige and Skoog micronutrient salts (Sigma), 3 mM CaCl_2_, 1.5 mM MgSO_4_, 1.25 mM KH_2_PO_4_, 5 mM KCl, 2 mM MES, pH 5.7. Effects of N limitation on plant growth in liquid condition were done as described [71]. For nitrogen-limitation treatment of adult plants, plants were grown hydroponically on vermiculite and perlite (1∶1, v/v) with the half Hoagland hydroponic Solution. When the plants started bolting at about 23 DAS, the solution was replaced with either the same nitrogen-rich solution as the control or nitrogen-free solution, and plants were grown for 5 days before the rosette leaves and stems were harvest respectively.

For C-limiting experiments, seedlings grown in a short-day photoperiod (8 h/16 h) for 9 days were transferred to soil and grown for 33 more days. The plants were then transferred to continuous darkness for 9 days, and then returned to the short-day photoperiod for a 25-day recovery. Two independent experiments were done; results from one were shown here.

### Quantification of Nitrogen, Soluble Sugar and Proteins

The juvenile and adult rosette leaves and the stems of the seedlings after 5-day N-limitation treatment were collected separately and rapidly frozen in liquid N_2_ and then stored at −80°C until analysis. Total leaf nitrogen was determined by Kjeldahl method [72]. Total proteins were extracted in extraction buffer as described [65], and the homogenates were centrifuged at 12,000 rpm for 15 min at 4°C. Protein concentrations of the supernatants were determined using the Bradford method [70]. Soluble sugar contents were determined as described [73]. Data shown are representative of at least three independent experiments; each includes three technical repeats with ten seedlings.

## Supporting Information

Figure S1 Overall structure of GmATG8c, HsGABARAP, ScATG8 and TbATG8. Image colored by rainbow N → C terminus.
(TIF)

Figure S2 Immunoblot analysis of the purified GmATG8c and protein extracts from one representative transgenic line (L-4).
(TIF)

Figure S3 Total contents of nitrogen, protein, and soluble sugar in rosette leaves and stems. Wild-type and *35S:GmATG8c* plants were grown hydroponically in half Hoagland’s until bolting (23 days after sowing), and then maintained in either the same nitrogen-rich solution (+N) or transferred to nitrogen-free solution (−N) for another 5 days. Total nitrogen (A), protein (B) and soluble sugar contents (C) in the rosette leaves and stem were measured. NS: N sufficient, ND: N deficient.*, p<0.05 (t-test); significant difference from the wild-type (WT).
(TIF)

Figure S4 Inflorescences of 7-week-old wild-type and transgenic plants. Ectopic expression of *GmATG8c* extended the period of flowering in Arabidopsis. White arrows indicate the flowers. WT: the wild-type; OE: *35S:GmATG8c* transgenic plants.
(TIF)

Figure S5 Heterologous expression of *GmATG8c* promotes growth and fruit setting in Tomato. A, Six-week-old wild-type (WT) and *35S:GmATG8c* transgenic tomato. B, Growth curves of the plant height of the wild-type (WT) and *35S:GmATG8c* tomato. C and D, Side views of eleven-week-old (C) and twelve-week-old (D) wild-type (WT) and *35S:GmATG8c* tomato.
(TIF)

Table S1 Genbank Accession Numbers Associated with soybean (*Glycine max*, Gm), *Physcomitrella patens* (Pp), *Selaginella moellendorffii* (Sm) and *Ostreococcus* lucimarinus (Ol) ATG8s.
(DOC)

Table S2 Primers used in this study.
(DOC)

Table S3 Rosette radius of the wild-type and 35S:GmATG8c lines without (+N) and with (−N) 5 days of nitrogen starvation. Data shown are the means ±SD of one representative biological replicate (n = 21) out of three. *, p<0.05 (t-test); significant difference from the wild-type (WT).
(DOC)

Table S4 The flowering time and the fruit setting of the wild-type and *35S:GmATG8c* transgenic tomato. *, p<0.05 (t-test); significant difference from the wild-type (WT), n = 21.
(DOC)

Methods S1 Description of the generation of the *35S:GmATG8c* transgenic tomato in this study.
(DOC)
