# Supplementary figures and images for: Heterologous Expression of ATG8c from Soybean Confers Tolerance to Nitrogen Deficiency and Increases Yield in Arabidopsis
Source: PLoS One. 2012 May 22;7(5):e37217. doi: 10.1371/journal.pone.0037217 (PMC3358335; doi:10.1371/journal.pone.0037217)

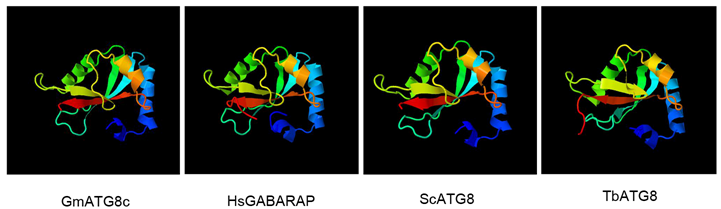

Supplement: Figure S1 — Overall structure of GmATG8c, HsGABARAP, ScATG8 and TbATG8. Image colored by rainbow N → C terminus. (TIF) [file pone.0037217.s001.tif]

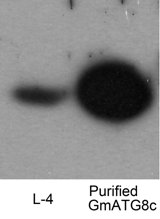

Supplement: Figure S2 — Immunoblot analysis of the purified GmATG8c and protein extracts from one representative transgenic line (L-4). (TIF) [file pone.0037217.s002.tif]

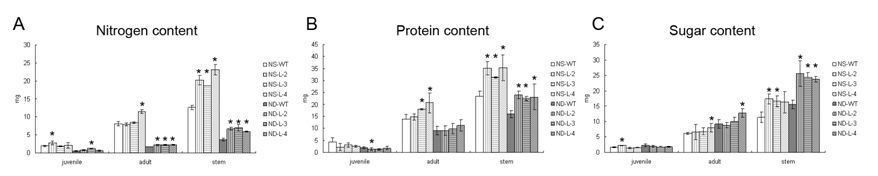

Supplement: Figure S3 — Total contents of nitrogen, protein, and soluble sugar in rosette leaves and stems. Wild-type and 35S:GmATG8c plants were grown hydroponically in half Hoagland’s until bolting (23 days after sowing), and then maintained in either the same nitrogen-rich solution (+N) or transferred to nitrogen-free solution (−N) for another 5 days. Total nitrogen (A), protein (B) and soluble sugar contents (C) in the rosette leaves and stem were measured. NS: N sufficient, ND: N deficient.*, p<0.05 (t-test); significant difference from the wild-type (WT). (TIF) [file pone.0037217.s003.tif]

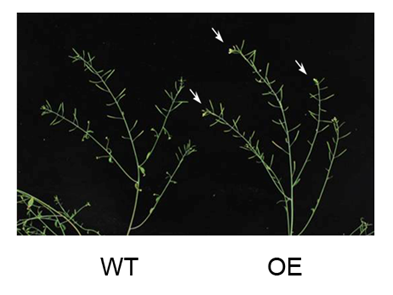

Supplement: Figure S4 — Inflorescences of 7-week-old wild-type and transgenic plants. Ectopic expression of GmATG8c extended the period of flowering in Arabidopsis. White arrows indicate the flowers. WT: the wild-type; OE: 35S:GmATG8c transgenic plants. (TIF) [file pone.0037217.s004.tif]

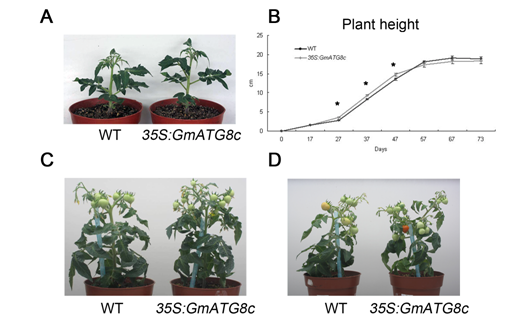

Supplement: Figure S5 — Heterologous expression of GmATG8c promotes growth and fruit setting in Tomato. A, Six-week-old wild-type (WT) and 35S:GmATG8c transgenic tomato. B, Growth curves of the plant height of the wild-type (WT) and 35S:GmATG8c tomato. C and D, Side views of eleven-week-old (C) and twelve-week-old (D) wild-type (WT) and 35S:GmATG8c tomato. (TIF) [file pone.0037217.s005.tif]
