# Supplementary material for: Heterologous Expression of ATG8c from Soybean Confers Tolerance to Nitrogen Deficiency and Increases Yield in Arabidopsis
Source: PLoS One. 2012 May 22;7(5):e37217. doi: 10.1371/journal.pone.0037217 (PMC3358335; doi:10.1371/journal.pone.0037217)
Supplement: Table S1 — Genbank Accession Numbers Associated with soybean (Glycine max, Gm), Physcomitrella patens (Pp), Selaginella moellendorffii (Sm) and Ostreococcus lucimarinus (Ol) ATG8s. (DOC) [file pone.0037217.s006.doc]

**Supplemental Table S1.** Genbank Accession Numbers Associated with *Glycine max* (Gm), *Physcomitrella patens* (Pp), *Selaginella moellendorffii* (Sm) and *Ostreococcus lucimarinus* (Ol) *ATG8s*.

| Gene | Phytozome ID | GenBank Accession No. | No. of Amino  Acid Residues | Identity to Yeast |
| --- | --- | --- | --- | --- |
| *GmATG8a* | Glyma17g01650.1 | ACU13796 | 119 | 71.43% |
| *GmATG8b* | Glyma07g39090.1 | ACU14633 | 129 | 67.44% |
| *GmATG8c* | Glyma09g00630.1 | ACU17086 | 119 | 70.59% |
| *GmATG8d* | Glyma12g10510.1 | BAH22449 | 120 | 71.67% |
| *GmATG8e* | Glyma06g46270.1 | ACU15101 | 120 | 71.67% |
| *GmATG8f* | Glyma05g04540.1 | ACU19559 | 134 | 64.18% |
| *GmATG8g* | Glyma17g14970.1 | ACU13862 | 128 | 66.41% |
| *GmATG8h* | Glyma10g01220.1 | ACU16419 | 122 | 48.36% |
| *GmATG8i* | Glyma02g01180.1 | BAH22448 | 122 | 47.54% |
| *GmATG8j* | Glyma11g03460.1 |  | 154 | 55.84% |
| *GmATG8k* | Glyma01g41910.1 | ACU19611 | 123 | 68.29% |
| *PpATG8a* | Pp1s209_114V6 | XP_001776702 | 123 | 70.73% |
| *PpATG8b* | Pp1s209_115V6 | XP_001776703 | 122 | 70.49% |
| *PpATG8c* | Pp1s209_118V6 |  | 122 | 71.31% |
| *PpATG8d* | Pp1s26_103V6 | XP_001757121 | 122 | 72.13% |
| *PpATG8e* | Pp1s249_49V6 | XP_001779032 | 125 | 69.60% |
| *PpATG8f* | Pp1s249_51V6 |  | 115 | 66.94% |
| *SmATG8a* | Smoellindorffii|15420269_locus|166976 | XP_002964670 | 121 | 66.94% |
| *SmATG8b* | Smoellindorffii|15405081_locus|109593 | XP_002978705 | 126 | 70.63% |
| *SmATG8c* | Smoellindorffii|15422493_locus|233421 | XP_002979699 | 118 | 55.83% |
| *OlATG8c* |  | XP_001415651 | 133 | 64.66% |
