# Supplementary material for: Heterologous Expression of ATG8c from Soybean Confers Tolerance to Nitrogen Deficiency and Increases Yield in Arabidopsis
Source: PLoS One. 2012 May 22;7(5):e37217. doi: 10.1371/journal.pone.0037217 (PMC3358335; doi:10.1371/journal.pone.0037217)
Supplement: Table S2 — Primers used in this study. (DOC) [file pone.0037217.s007.doc]

**Supplemental Table S2.** Pimers Used in this study.

| Category | Gene | Primer | Sequence (5’-3’) |
| --- | --- | --- | --- |
| Gene cloning | *GmATG8c* | 35S GmATG8c-F | ATTTCGCCATGGCCAAAACCTCCTTCAAGCTTC |
|  |  | 35S GmATG8c-R | GATTTGGGTCACCTATTTACAGAAGTTAACATGCATTATGC |
| Yeast complementation | *GmATG8c* | ycGmATG8c-F | ACTAGTATGGCCAAAACCTCCTTC |
|  |  | ycGmATG8c-R | AGATCTCTAATGGGATCCGAAGGTG |
|  | *ScADH1 promoter* | ScADH1p-F | GAATTCTGCATGCCTGCAGGTCGAG |
|  |  | ScADH1p-R | ACTAGTTTGGAGTTGATTGTATGC |
| Real-time RT-PCR | *GmATG8a* | qGmATG8a-F | CTCAACCTAATTCCCCATTCATCCA |
|  |  | qGmATG8a-R | TGTCTTCTTTCCAAGGGATGTTCAA |
|  | *GmATG8b* | qGmATG8b-F | ATGTTTTCTAATGTTTCTCTGTTTG |
|  |  | qGmATG8b-R | CAGACTCAGCCTGCCTTCTTTCCAA |
|  | *GmATG8c* | qGmATG8c-F | AACAGAACAACATCAAACCAAACCC |
|  |  | qGmATG8c-R | GAGGCTATGTCTCTGTCTGAGTTCG |
|  | *GmATG8d* | qGmATG8d-F | GGGAGTTTCCTCATTTTTCTTCTTC |
|  |  | qGmATG8d-R | TCAGAAGAGAAATGAGAATGAAGAG |
|  | *GmATG8e* | qGmATG8e-F | TTTTCTGTTTCTCCTTTTTTGGTTC |
|  |  | qGmATG8e-R | AAGGTTAAAAGAAAAAAAGAAGGCA |
|  | *GmATG8f* | qGmATG8f-F | GCAATCAGAGGTTTTTATCTTTTCA |
|  |  | qGmATG8f-R | AATTAAAAAGAGCCGAAGTTTCAGG |
|  | *GmATG8g* | qGmATG8g-F | GTAGGTCCATCTTCAATCTTCATCT |
|  |  | qGmATG8g-R | TTAGAAATAAAAAAAAAGGGGGGCA |
|  | *GmATG8h* | qGmATG8h-F | AACTGTTAAACTCTGTACACTATTG |
|  |  | qGmATG8h-R | TAAACCATACAGGTGATACAACAAA |
|  | *GmATG8i* | qGmATG8i-F | GTAAAATATTAAACTCTGTACACTA |
|  |  | qGmATG8i-R | ATAGTGATACATTAAACCATACAAC |
|  | *GmATG8j* | qGmATG8j-F | TTACTTCTCTCTTGGTTTTCTTGAT |
|  |  | qGmATG8j-R | GCAAAACGTATATACCAAGATCATG |
|  | *GmATG8k* | qGmATG8k-F | GTTACTTCTCTCTTGGTTTTCTTCA |
|  |  | qGmATG8k-R | TTAAGACAAGCAAGAACATCAAAGT |
|  | *AtATG3* | qAtATG3-F | TCATCCACACTTGCCTGGTA |
|  |  | qAtATG3-R | CCGAGATCAAAGTCCATTGTG |
|  | *AtATG10* | qAtATG10-F | CCCTGTGGGACTGAGGACTGGA |
|  |  | qAtATG10-R | AGACCAACCACCTGCCCGACA |
|  | *AtATG12a* | qAtATG12a-F | AGTGCTTTCTCGCCAAACCCTGA |
|  |  | qAtATG12a-R | GTCTTCAGTTTTAGCCCCATGCCA |
|  | *AtATG18a* | qAtATG18a-F | GGCACTGACCGTGGCTTCCG |
|  |  | qAtATG18a-R | ACGACTGCAACACCACCGCC |
|  | *TIP41-LIKE* | qTip-F | GTATGAAGATGAACTGGCTGACAAT |
|  |  | qTip-R | ATCAACTCTCAGCCAAAATCGCAAG |
