# Supplementary material for: Heterologous Expression of ATG8c from Soybean Confers Tolerance to Nitrogen Deficiency and Increases Yield in Arabidopsis
Source: PLoS One. 2012 May 22;7(5):e37217. doi: 10.1371/journal.pone.0037217 (PMC3358335; doi:10.1371/journal.pone.0037217)
Supplement: Table S3 — Rosette radius of the wild-type and 35S:GmATG8c lines without (+N) and with (−N) 5 days of nitrogen starvation. Data shown are the means ±SD of one representative biological replicate (n = 21) out of three. *, p<0.05 (t-test); significant difference from the wild-type (WT). (DOC) [file pone.0037217.s008.doc]

**Supplemental Table S3.** Rosette radius of the wild-type and *35S:GmATG8c* lines without (+N) and with (-N) 5 days of nitrogen starvation.

|  | WT (cm) | L-2 (cm) | L-3 (cm) | L-4 (cm) |
| --- | --- | --- | --- | --- |
| +N | 3.62±0.29 | 3.93±0.37* | 4.00±0.38* | 4.07±0.39* |
| -N | 2.97±0.27 | 3.67±0.56* | 3.81±0.48* | 3.87±0.44* |

Data shown are the means ±SD of one representative biological replicate (n = 21) out of three. *, p < 0.05 (t-test); significant difference from the wild-type (WT).
