# Supplementary material for: Heterologous Expression of ATG8c from Soybean Confers Tolerance to Nitrogen Deficiency and Increases Yield in Arabidopsis
Source: PLoS One. 2012 May 22;7(5):e37217. doi: 10.1371/journal.pone.0037217 (PMC3358335; doi:10.1371/journal.pone.0037217)
Supplement: Table S4 — The flowering time and the fruit setting of the wild-type and 35S:GmATG8c transgenic tomato. *, p<0.05 (t-test); significant difference from the wild-type (WT), n = 21. (DOC) [file pone.0037217.s009.doc]

**Supplemental Table S4.** The flowering time and the fruit setting of the wild-type and *35S*:GmATG8c transgenic tomato.

|  | WT | 35S:GmATG8c |
| --- | --- | --- |
| Flowering time (d) | 50.88±2.39 | 47.44±1.31 |
| Total number of fruits | 21.31±1.05 | 30.37±1.65* |
| Rate of Fruit-set | 60.57±2.8% | 72.43±3.9%* |

*, p < 0.05 (t-test); significant difference from the wild-type (WT), n = 21.
