# Supplementary material for: Heterologous Expression of ATG8c from Soybean Confers Tolerance to Nitrogen Deficiency and Increases Yield in Arabidopsis
Source: PLoS One. 2012 May 22;7(5):e37217. doi: 10.1371/journal.pone.0037217 (PMC3358335; doi:10.1371/journal.pone.0037217)
Supplement: Methods S1 — Description of the generation of the 35S:GmATG8c transgenic tomato in this study. (DOC) [file pone.0037217.s010.doc]

**Methods S1**

Tomato (*Solanum lycopersicum*) cv. Micro-Tom was also used for heterologous expression of *GmATG8c*. Micro-Tom was transformed with construct *35S:GmATG8c* with a protocol derived from the method described before . Briefly, cotyledons of 7-day-old seedlings were cut at the tip near the petiole, laid on a plate, and preincubated for 24 h at 25 ^o^C under low light conditions. The concentration of Agrobacterium strain LBA 4404 used for co-cultivation ranged from 5*10^-7^ to 9*10^-7^ cfu ml^-1^, corresponding to an OD ranging from 0.4 to 0.5. Co-cultivation was carried out under the same conditions as preincubation for 48 h. Subsequently, the cotyledons were transferred to 2Z medium containing 100 μg ml^-1^ kanamycin and 400 μg ml^-1^ carbencillin for 3-4 weeks, then transferred to 1Z medium with 200 μg ml^-1^ carbenicillin for 2-3 weeks. Shoots were then excised from the cotyledons and transferred to a rooting medium supplemented with 2 μg ml^-1^ IBA, 50 μg ml^-1^ kanamycin, and 100 μg ml^-1^ carbenicillin. Plantlets with roots appeared after 1-3 weeks and then transferred to the growth room at 24 °C under a long-day photoperiod (16 h/8 h).

The wild-type and transgenic tomato plants were grown in a growth room at 24 °C under a long-day photoperiod (16 h/8 h). Plant height and total number of fruits were measured and counted, and rates of fruit set was calculated as described .

**Supplemental References**

1. Meissner R, Jacobson Y, Melamed S, Levyatuv S, Shalev G, et al. (1997) A new model system for tomato genetics. The Plant Journal 12: 1465-1472.

2. Fillatti JJ, Kiser J, Rose R, Comai L (1987) Efficient Transfer of a Glyphosate Tolerance Gene into Tomato Using a Binary Agrobacterium Tumefaciens Vector. Nat Biotech 5: 726-730.

3. Sato S, Peet MM, Thomas JF (2000) Physiological factors limit fruit set of tomato (Lycopersicon esculentum Mill.) under chronic, mild heat stress. Plant Cell and Environment 23: 719-726.
